# The Tuberculosis Cure?

**Published:** 1921-02-26

**Authors:** 


### The Tuberculosis Cure?

Dr. Addison, speaking at Exeter, on February 18, made the following statement:

Reference has been made in the papers to-day to the alleged discovery of a serum which is said to be a cure for the terrible white scourge of tuberculosis, and it is reported that the Ministry of Health has sent a special medical officer to Geneva to investigate this cure. This is not the case. The idea must have arisen through some misapprehension as to the actual steps which have been taken, as a result of which such action is not necessary at present. It is, of course, the habitual practice of the Ministry of Health to watch any new contributions to medical science, and I have already made careful inquiries respecting this alleged cure.

But it is most important, having in view the fact that widely published statements may raise unfounded hopes in the hearts of consumptives, that I should state, as clearly as possible, that in the case of this supposed remedy much further investigation will be necessary. I understand that a scientific report on the method of treatment has been presented to the French Academy of Science. When this has been published, and when supplies of the serum are available, I shall gladly facilitate and co-operate in an exhaustive examination of its efficacy under the supervision of experts.

It is, therefore, of importance for all to know that no supply of serum for investigation is at present available in this country. It is not expected to be available for some months to come, according to our present information.

The Ministry of Health, as part of its routine duty, and in co-operation with the Medical Research Council and the International Health Office, are, of course, fully alive to the importance of keeping in the closest possible touch with all helpful suggestions for improvements in the treatment of tuberculosis; but we must remember that the results of lengthy and varied tests and of clinical experience must be obtained in this case, as in all others, before we should be justified in recommending it for general use.

